# Mesenchymal stem cell–conditioned medium prevents radiation-induced liver injury by inhibiting inflammation and protecting sinusoidal endothelial cells

**DOI:** 10.1093/jrr/rrv026

**Published:** 2015-06-11

**Authors:** Yi-Xing Chen, Zhao-Chong Zeng, Jing Sun, Hai-Ying Zeng, Yan- Huang, Zhen-Yu Zhang

**Affiliations:** 1 Department of Radiation Oncology, Zhongshan Hospital, Fudan University, Shanghai, 200032, China; 2 Department of Pathology, Zhongshan Hospital, Fudan University, Shanghai, 200032, China

**Keywords:** sinusoidal endothelial cell, radiation-induced liver injury, mesenchymal stem cell, apoptosis

## Abstract

Current management of radiation-induced liver injury is limited. Sinusoidal endothelial cell (SEC) apoptosis and inflammation are considered to be initiating events in hepatic damage. We hypothesized that mesenchymal stem cells (MSCs) possess anti-apoptotic and anti-inflammatory actions during hepatic irradiation, acting via paracrine mechanisms. This study aims to examine whether MSC-derived bioactive components are protective against radiation-induced liver injury in rats. MSC-conditioned medium (MSC-CM) was generated from rat bone marrow–derived MSCs. The effect of MSC-CM on the viability of irradiated SECs was examined by flow cytometric analysis. Activation of the Akt and ERK pathways was analyzed by western blot. MSC-CM was also delivered to Sprague–Dawley rats immediately before receiving liver irradiation, followed by testing for pathological features, changes in serum hyaluronic acid, ALT, and inflammatory cytokine levels, and liver cell apoptosis. MSC-CM enhanced the viability of irradiated SECs *in vitro* and induced Akt and ERK phosphorylation in these cells. Infusion of MSC-CM immediately before liver irradiation provided a significant anti-apoptotic effect on SECs and improved the histopathological features of injury in the irradiated liver. MSC-CM also reduced the secretion and expression of inflammatory cytokines and increased the expression of anti-inflammatory cytokines. MSC-derived bioactive components could be a novel therapeutic approach for treating radiation-induced liver injury.

## INTRODUCTION

Hepatocellular carcinoma (HCC) is the fifth most common malignancy worldwide and accounts for nearly 10% of cancer deaths annually [1]. However, the majority of HCC patients present with advanced-stage disease. In these patients, radiotherapy (RT) is one of the major methods for palliative treatment. However, the major limitation of RT is the high risk of potentially lethal liver injury, later termed radiation-induced liver disease (RILD) [2, 3]. Current RILD management is mainly supportive, and the mortality rates exceed 75% [4, 5]. Developing new approaches to protect the liver from injury after irradiation is necessary and important.

The liver is a highly radiosensitive organ, and the threshold dose for whole-liver irradiation is reportedly between 20 and 30 Gy [6]. However, hepatocytes are considered more radioresistant than other cells [7–11]. Thus, hepatocytes are not the direct and initial target in radiation-induced liver injury. RILD is characterized by early veno-occlusive disease (VOD) [12, 13], and sinusoidal endothelial cell (SEC) injury has been traditionally postulated as the initiating lesion of VOD in RILD [13]. Yamanouchi *et al.* [14] reported that the number of apoptotic SECs increased significantly 6 h after 30 Gy of liver irradiation in a rat model; by contrast, apoptotic hepatocytes were only occasionally seen. SEC injury results in microcirculatory blood flow disturbances and secondary injury to hepatocytes, causing liver dysfunction. Inflammation is also important in RILD development. The inflammatory cytokines tumor necrosis factor α (TNF-α), interleukin 1β (IL-β), and IL-6, as well as numerous chemokines, have been implicated in early-phase RILD development [15–18]. Therefore, SECs are the primary targets in liver radiation, and inflammation enhances this process.

Mesenchymal stem cells (MSCs) show significant potential for clinical utility, due to their convenient isolation and culture, low immunogenicity, regenerative and multiple differentiation abilities, and potent anti-apoptotic and immunosuppressive effects [19]. Recent studies have shown that MSCs can ameliorate tissue injury resulting from radiation, including radiation-induced lung injury and radiation-induced intestinal injury [20–24]. The mechanisms of MSC action responsible for preventing radiation-induced tissue damage may involve paracrine secretory mechanisms [25]. Paracrine factors expressed by MSCs include different types of cytokines, such as anti-apoptotic factors, growth factors, and both anti-inflammatory and inflammatory factors [26–30]. Chang *et al.* [24] reported that bone marrow transplantation rescued intestinal mucosa after whole-body irradiation by secreting cytokines that enhance angiogenesis and chemotaxis. Therefore, systemic infusion of MSC-conditioned medium (MSC-CM) into recipients with irradiated livers may be an effective and appealing approach to reducing liver injury.

This study investigated whether systemic infusion of MSC-CM could protect SECs after radiation, which might eventually mitigate RILD. We injected MSC-CM into rats before liver irradiation. Terminal nucleotidyl transferase–mediated dUTP nick end labeling (TUNEL) was used to assess the apoptosis in liver tissue. Using an *in vitro* assay, we demonstrate that MSC secretory products have a direct inhibitory effect on SEC apoptosis and provide evidence that the Akt and ERK pathways may mediate this effect.

## MATERIALS AND METHODS

### Animals

Nine-week-old inbred male Sprague–Dawley rats (Animal Center of Zhongshan Hospital, Fudan University, Shanghai, China) were used in this study. Rats were maintained under controlled conditions (24 ± 2°C temperature, 40–70% relative humidity, and 12-h light/12-h dark cycle) and given a normal laboratory diet and water *ad libitum*, in accordance with the criteria of the Guide for the Care and Use of Laboratory Animals of Fudan University.

### Cell culture and MSC-CM preparation

Rat liver SECs were purchased from PriCells Company (Wuhan, China). SECs were seeded in low DMEM medium (Invitrogen, Cergy Pontoise, France) supplemented with 15% fetal bovine serum (Gibco-BRL, Grand Island, NY), 10 ng/ml epidermal growth factor, 2 ng/ml hydrocortisone, 2 mmol/l L-glutamine, 100 units/ml penicillin and 100 ng/ml streptomycin, referred to as EC complete medium.

Rat bone marrow MSCs were isolated, cultured and identified as previously described [31]. To generate MSC-CM, passage 3–4 MSC cells were grown to 80–90% confluence in 25-cm^2^ flasks, washed thoroughly, and cultured in serum-free low-glucose DMEM for 24 h. MSC-CM was collected by filtering through a 50-μm mesh.

### Experimental design and irradiation procedure

For *in vitro* experiments, irradiated SECs (15 Gy) were seeded in the bottom of 6-well plates and incubated with DMEM, or MSC-CM (2 ml, MSC-CM was collected from 1×105 MSCs) for 2 h. After incubation, apoptosis in SECs was examined by fluorescence-activated cell sorting (FACS). When confirming activation of the Akt and ERK pathways, PI3 K/Akt inhibitor (LY294002) and MEK/ERK inhibitor (U0126) were respectively added 2.5 h before irradiation, and the apoptotic SECs were counted by FACS.

For *in vivo* experiments, MSC-CM was concentrated using an Amicon Ultra Centrifugal Filter Device (Millipore). Animals were allocated into one of three experimental groups: (i) Control group, (ii) RT + DMEM group, and (iii) MSC-CM + RT group. Because the effect of MSC-CM alone on normal liver was not the focus of this study, we excluded MSC-CM with normal liver in the Control group. As described previously [32], rats were anesthetized, and a 20-Gy dose of X-rays was delivered to rats' livers in a single fraction using a linear accelerator (Oncor; Siemens, Munich, Germany). Organs near the liver were protected by lead shielding. A total of 1.0 ml MSC-CM (equal numbers of 1 × 10^6^ MSCs) or medium was injected into the penile vein immediately before liver irradiation. Liver tissues and blood samples were collected at different times. Parts of the tissues were fixed, and the remaining tissues and serum samples were stored at −80°C.

### Apoptosis determination

For *in vitro* studies, apoptosis was measured by flow cytometric analysis. SECs were pretreated with MSC-CM or DMEM for 2 h prior to 15-Gy irradiation. Cells were harvested at 24 h after irradiation. Apoptosis was determined using an Annexin V-FITC/7-oxaloacetic acid (OAA) fluorescent cell staining kit (KeyGEN, BioTECH, Nanjing, China), according to the manufacturer's instructions.

For *in vivo* apoptosis studies, liver tissue samples were formalin-fixed and paraffin-embedded, and 4-µm sections were stained with a TUNEL protocol (KeyGEN, BioTECH, Nanjing, China).

### Serum analysis

Serum levels of TNF-α, IL1-β, IL-6, IL-10 and hyaluronic acid (HA) were quantified using enzyme-linked immunosorbent assays (ELISAs) as per the manufacturer's instructions (R&D Systems Inc., Minneapolis, MN). Alanine aminotransferase (ALT) was measured using an enzymatic assay (Biotron Diagnostics, Inc., Hemet, CA), according to the manufacturer's instructions.

### Real-Time quantitative Reverse Transcription-PCR

Total RNA was isolated from the liver tissue was used for real-time quantitative reverse transcription-polymerase chain reaction **(**RT-qPCR**)** (Toyobo, Osaka, Japan) using the QuantiTect SYBR Green Reverse Transcriptase-Polymerase Chain Reaction kit (Qiagen, Valencia, CA). The sequences of the specific primers for TNF-α, IL1-β, IL-6, IL-10 and β-actin are shown in Supplemental Table e1.

### Liver histology

Formalin-fixed, paraffin-embedded liver tissues were sectioned (4-µm thick), and sections were stained with H&E and observed by light microscopy.

### Protein isolation and western blot

SECs were grown to confluence in six-well culture plates, and media were replaced with serum-free DMEM medium (2 ml/well). After 2 h, media were replaced with MSC-CM i, and SECs were incubated for an additional 45 min before harvesting for cellular protein analysis. For western blot analysis, equal amounts of SEC cellular proteins were resolved by 10% SDS–PAGE and transferred to PVDF membranes. Antibodies against the following proteins used to probe membranes were used as primary antibodies: phospho-Akt and phospho-ERK1/2 (all from Cell Signaling, Beverly, MA).

### Statistics

Data are expressed as means ± standard deviation (SD). Comparisons among multiple groups were performed by non-parametric analysis of variance (ANOVA). Differences were considered to be statistically significant when *P* < 0.05.

## RESULTS

### MSC-CM inhibits radiation-induced SEC apoptosis *in vitro*

**Fig. 1.**
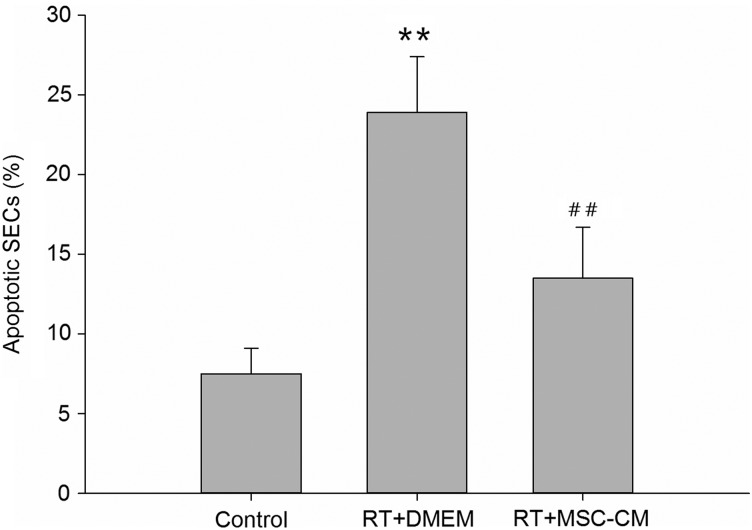
Anti-apoptotic effect of MSC-CM on SECs *in vitro*. Pretreatment with MSC-CM decreased the number of apoptotic SECs. Apoptosis was determined by fluorescence-activated cell sorting (FACS) analysis. Data are shown as means ± SD. **P < 0.01 (DMEM group versus control group). ^##^*P* < 0.01 (MSC-CM group versus DMEM group).

To determine whether secretory products in MSC-CM decrease radiation-induced SEC apoptosis, we performed (FACS) analyses in combination with annexin V staining. Pretreatment with MSC-CM decreased the number of apoptotic SECs 24 h after 15 Gy irradiation to 13.5 ± 2.9% of the total SEC population versus 23.6 ± 3.2% when incubated in DMEM only (*P* < 0.01; Fig. 1). The results confirm that MSC-CM therapy effectively reduces the SEC apoptosis that is induced by irradiation.

### MSC-CM inhibits radiation-induced SEC apoptosis *in vivo*

**Fig. 2.**
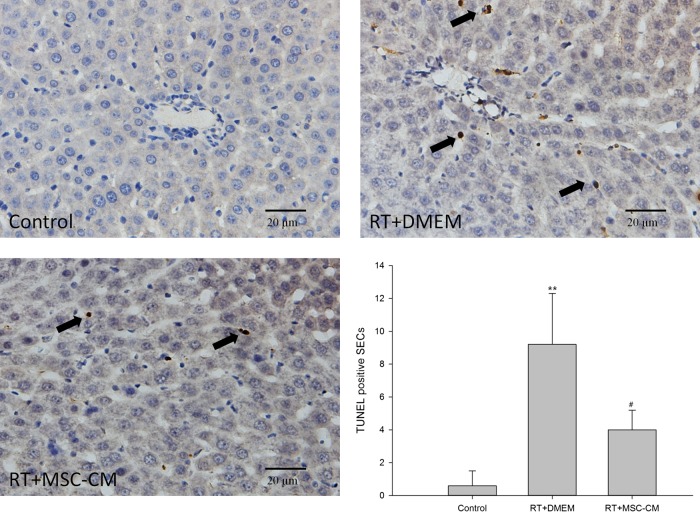
Effect of MSC-CM pretreatment on radiation-induced SEC apoptosis in rat liver. Irradiation initiated SEC apoptosis in rat liver SECs, which was significantly decreased by a pre-RT intravenous injection of MSC-CM. Five randomly selected areas were viewed by light microscopy (×400 total magnification) to enumerate apoptotic SECs positive for terminal nucleotidyl transferase-mediated  nick end labeling (TUNEL) staining. Arrows indicate apoptotic SECs. Data are shown as means ± SD. **P < 0.01 (DMEM group versus control group). ^#^*P* < 0.05 (MSC-CM group versus DMEM group).

We used the TUNEL assay to evaluate SEC apoptosis in liver sections generated in an irradiated rat model. Liver irradiation significantly increased the number of apoptotic SECs from 6 h after RT compared with the non-RT control group (Fig. 2). There were 9.2 ± 3.1 TUNEL-positive SECs per high-power field, compared with 0.6 ± 0.9 cells per high-power field in the control group (*P* < 0.05) at 6 h. Treatment with MSC-CM significantly reduced the number of apoptotic SECs induced by RT (4.0 ± 1.2, *P* < 0.05). These data demonstrate that MSC-CM specifically inhibits radiation-induced SEC apoptosis *in vivo*. Unlike SECs, the number of TUNEL-positive hepatocytes was few in irradiated rat liver sections.

### Serum HA and ALT levels after radiation and MSC-CM therapy *in vivo*

**Fig. 3.**
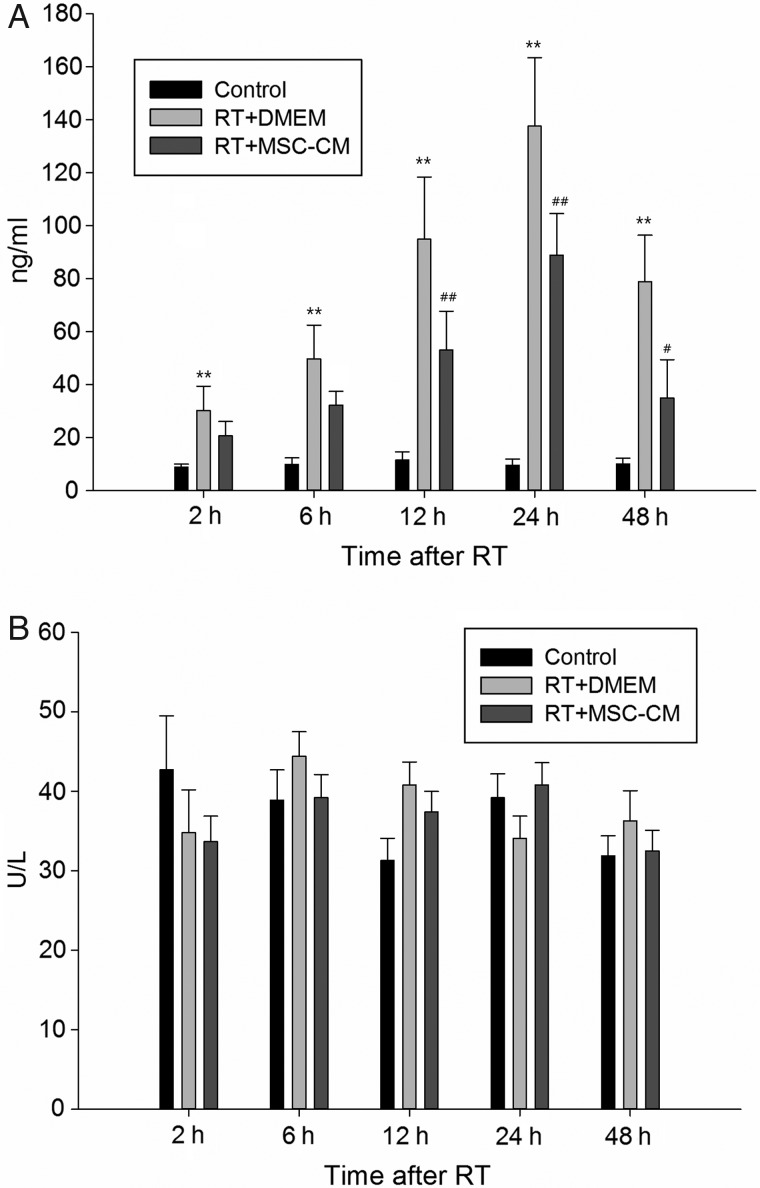
Effect of liver irradiation on serum hyaluronic acid (HA) and ALT levels in rats. (A) Circulating HA levels (SEC-derived) were time-dependently increased in rats after liver irradiation versus non-irradiated controls, and this effect was decreased by intravenous infusion of MSC-CM. (B) Irradiation did not alter circulating ALT levels (hepatocyte-derived). Data are shown as means ± SD. **P < 0.01 (DMEM group versus control group). ^##^*P* < 0.01 and ^#^*P* < 0.05 (MSC-CM group versus DMEM group).

Elevated serum HA and ALT levels are markers of SEC and hepatocyte injury, respectively. Serum HA levels in liver-irradiated rats were significantly increased at 6 h after RT compared with the non-RT control group, and this effect was maximal at 24 h (control group, 9.6 ± 2.3 ng/ml versus RT group, 137.6 ± 25.8 ng/ml; *P* < 0.01) (Fig. 3A). Pretreatment with systemic MSC-CM significantly alleviated SEC damage in liver-irradiated rats, evidenced by a significant reduction in serum HA levels to 88.9 ± 15.7 ng/ml at 24 h (Fig. 3A). However, serum ALT serum did not significantly increase throughout the 48-h experiment (Fig. 3B). These data together indicate that the acute effect of RT on hepatocyte death is minimal; however, SECs are much more sensitive to radiation-induced damage and killing, and soluble factors in MSC-CM have a protective effect on these cells.

### MSC-CM therapy modulates the inflammatory response to liver irradiation *in vivo*

**Fig. 4.**
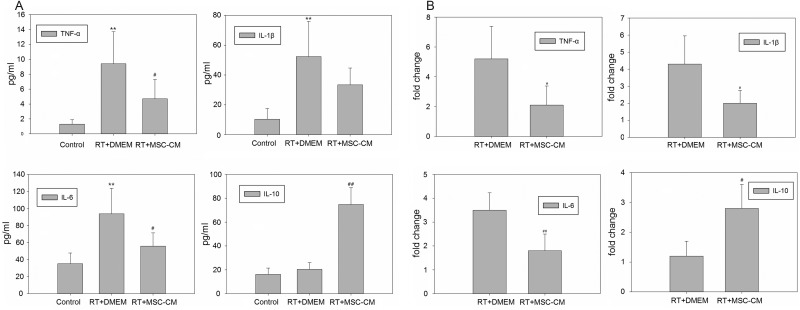
Expression levels of inflammatory and anti-inflammatory cytokines in peripheral blood and liver tissue. Samples were collected 6 h after liver irradiation. Enzyme-linked immunosorbent assay (ELISA) (A) and Real-Time quantitative Reverse Transcription-PCR (RT-qPCR) (B) analyses showed that TNF-α, IL-β and IL-6 were all significantly increased after RT, and these effects were partially decreased by MSC-CM administration. Expression of the anti-inflammatory cytokine IL-10 was significantly increased by MSC-CM treatment. Data are shown as means ± SD. **P < 0.01 (DMEM group versus control group). ^##^*P* < 0.01 and ^#^*P* < 0.05 (MSC-CM group versus DMEM group).

Inflammatory cytokines are known to be upregulated during liver injury [11]. Liver irradiation in rats significantly increased serum concentrations of the inflammatory cytokines TNF-α, IL-β and IL-6, and systemic MSC-CM treatment inhibited this response (*P* < 0.05 for TNF-α and IL-6, Fig. 4A). Conversely, levels of the anti-inflammatory cytokine IL-10 were increased in the MSC-CM treated group (*P* < 0.01). Real-time RT-qPCR was used to assess the local inflammatory reaction in liver tissue 6 h after liver irradiation (Fig. 4B). Compared with the radiation-only group, systemic MSC-CM administration significantly reduced the mRNA expression levels of TNF-α, IL-1β and IL-6, and increased IL-10 mRNA expression of IL-10 in irradiated rat livers (*P* < 0.05 for TNF-α and IL-10, *P* < 0.01 for IL-1β and IL-6). Taken together, these data demonstrate that intravenous infusion of MSC-secreted molecules can modulate local and systemic inflammatory responses associated with RILD.

### MSC-CM therapy improves liver histopathology

**Fig. 5.**
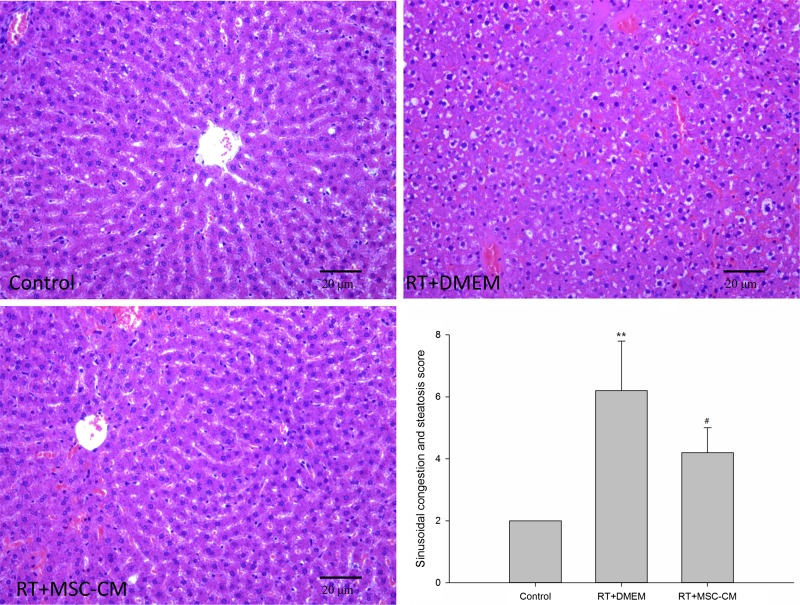
Effect of MSC-CM on radiation-induced liver injury. Liver histopathology showed radiation-induced sinusoidal congestion and hepatic steatosis at 4 weeks after RT (original magnification ×200). These features were improved by systemic MSC-CM administration. Data are shown as means ± SD. **P < 0.01 (DMEM group versus control group). ^#^*P* < 0.01 (MSC-CM group versus DMEM group).

Histological examination of liver biopsy specimens was used to assess long-term injury. Liver irradiation resulted in typical sinusoidal congestion and hepatic steatosis in otherwise normal livers; at 4 weeks after RT, the sinusoidal congestion and steatosis score for RT+DMEM group was 6.2 ± 1.6 (Fig. 5); however, these changes were much less pronounced in rats pretreated with MSC-CM (4.2 ± 0.8, *P* < 0.05).

### Effects of MSC-CM on Akt and ERK phosphorylation in SECs

**Fig. 6.**
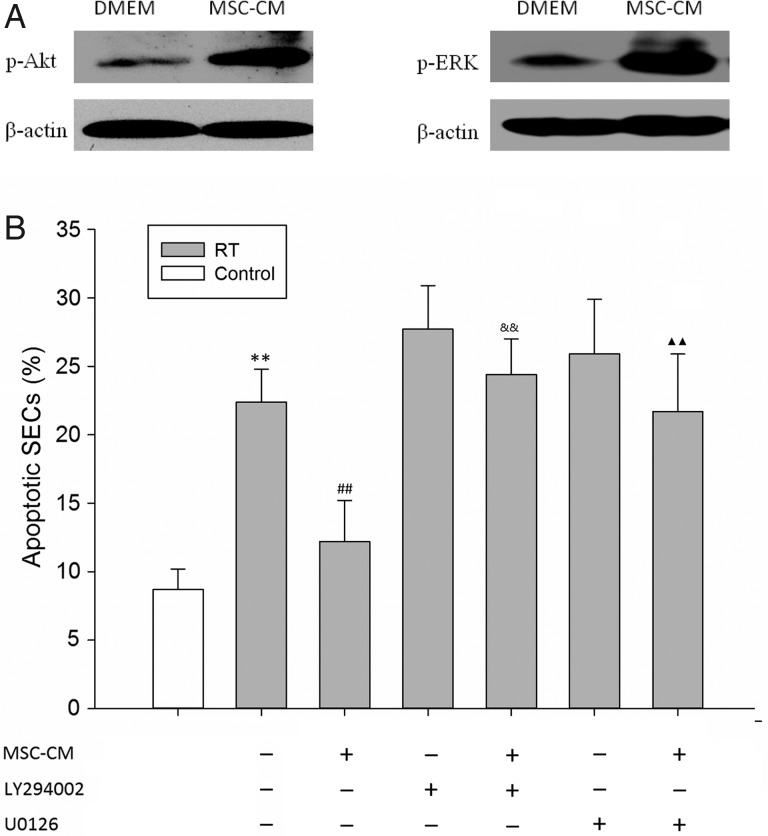
(A) Phospho-AKT and Phospho-ERK1/2 expression by western blot in SECs treated with or without MSC-CM for 45 min. (B) SECs were cultured in the presence of the PI3 K/Akt inhibitor (LY294002) and MEK/ERK inhibitor (U0126) with or without RT and MSC-CM, and cell apoptosis was evaluated by fluorescence-activated cell sorting (FACS) analysis. Data are shown as means ± SD. **P < 0.01 (DMEM group versus control group). ^##^*P* < 0.01 (MSC-CM group versus DMEM group). ^&&^*P* < 0.01 (MSC-CM group versus MSC-CM + LY294002 group). Two closed triangles: *P* < 0.01 (MSC-CM group versus MSC-CM + U0126 group).

Phosphorylation of the intracellular signaling molecules Akt and ERK is an important trigger of their anti-apoptosis and cell survival activities [33, 34]. Thus, we examined whether MSC-CM treatment affected Akt and ERK phosphorylation in cultured SECs. Treating serum-starved SECs with MSC-CM markedly induced Akt phosphorylation at 45 min (Fig. 6A). Similarly, MSC-CM induced ERK1/2 phosphorylation (Fig. 6B). To confirm the role of Akt and ERK activation in MSC-CM-mediated SEC survival, we used the Akt inhibitor (LY290004) and ERK inhibitor (U0126) to test the survival of SECs (Fig. 6C). LY290004 and U0126 inhibited MSC-CM–mediated radioprotection (apoptotic SECs, 24.4 ± 2.6% and 21.7 ± 4.2% after pretreatment with LY290004 and U0126, respectively).

## DISCUSSION

SEC apoptosis and inflammation are initial events in RILD. This study provided the first clear evidence that delivery of MSC secretory products has the potential to dramatically reduce SEC death in the acutely radiation-injured liver. *In vivo*, rat liver irradiation increased circulating HA levels, SEC apoptosis, and inflammatory cytokine expression, and all of these effects were inhibited by pre-RT treatment with intravenous MSC-CM injection, ultimately reducing RILD. These observations suggest that MSC-CM contains soluble factors that are capable of attenuating liver injury. Identification of these soluble factors secreted by MSCs may yield new therapeutic options for RILD.

We demonstrated that apoptosis after liver irradiation occurs in SECs, but not hepatocytes, in the early stage after RT. Radiation-induced SEC apoptosis, measured by TUNEL staining of liver sections, occurred within 6 h of irradiation. This effect is comparable with apoptosis of the small intestinal microvascular endothelium, which underwent apoptosis within 4 h of whole-body irradiation in mice [35]. However, fewer apoptotic SECs were observed in irradiated rat livers after pretreatment with intravenous MSC-CM infusion.

We also determined serum HA and ALT levels in rats with irradiated livers, as respective markers of SEC and hepatocyte integrity. HA is an unbranched glycosaminoglycan present in the extracellular matrix. More than 90% of circulating HA is removed by hepatic SECs via HA-specific receptor-mediated endocytosis [36]. Increased circulating HA serves as a biomarker of dysfunction of hepatic SECs following liver injury [37]. Elevated circulating ALT is a known marker of liver damage, specifically of hepatocytes. We found that serum HA levels reached peak values within 24 h of liver RT, but serum ALT levels did not significantly change during the 48 h post-irradiation. Reducing injury by maintaining SEC integrity can prevent further hepatocyte damage [38, 39]. Ma *et al.* reported that: *in vitro*, adipose-derived stem cells prevent apoptosis of freshly isolated liver SECs by secreting VEGF; *in vivo*, implanted syngeneic adipose-derived stem cells attenuated small-for-size liver graft injuries in a rat model [40].

In *in vitro* experiments, we also found that MSC-CM pretreatment can protect cultured SECs from radiation injury–induced apoptosis. This protective mechanism may involve activation of the Akt and ERK1/2 signaling pathways. The PI3 K/Akt and ERK1/2 pathways are activated by growth factors and are both important cell survival signaling pathways [35, 36]. In the present study, Akt and ERK1/2 phosphorylation was induced by MSC-CM, which suggests that the protective effect of MSC-CM may be mediated by Akt and ERK1/2 activation. This effect may be caused by some growth factors secreted by MSCs. It has been reported that MSCs express many diverse biological factors, including VEGF, βFGF, PDGF, IGF-1 and S1P, all of which have anti-apoptotic activity.

MSCs are emerging as a therapeutic modality for various inflammatory diseases because of their anti-inflammatory and immunomodulatory properties. It has been reported that MSC-CM reduces the inflammatory response in both ischemia–reperfusion injury and D-galactosamine–induced acute liver injury models [41, 42]. In other organ injury models, MSC transplantation downregulates inflammatory cytokines and upregulates anti-inflammatory cytokines such as IL-10 [43, 44]. In this study, we found that systemic MSC-CM administration decreased TNF-α, IL-β and IL-6, and increased IL-10 expression, both in peripheral blood and liver tissue after hepatic irradiation. Secretion of the anti-inflammatory cytokine IL-10 by MSCs has been investigated in several studies [23, 45, 46]. In a model of radiation-induced intestinal injury, Chang *et al.* also demonstrated a significantly increased IL-10 concentration in response to human MSC administration [23].

RILD has been confirmed as a VOD with sinusoidal congestion of the central portion of the liver lobules, and ultimate atrophy of hepatocytes and fibrosis of the pericentral lobule regions [3]. Our study suggests that sinusoidal congestion emerges at 4 weeks after liver RT, and systemic MSC-CM pretreatment before irradiation can mitigate this damage. We also detected the appearance of hepatic steatosis in addition to sinusoidal congestion in rat livers following 30-Gy radiation. Steatosis often occurs in alcoholic liver disease and other diverse toxin-mediated liver diseases, in which oxidative stress reactions mediate liver injury [47]. We speculate that hepatic steatosis might also result from radiation-induced liver toxicity, and additionally, administration of MSC-CM can improve radiation-induced hepatic steatosis.

Most reports have employed cell transplantation as the primary mode of MSC therapy. However, the liver is a large organ that is not suitable for local MSC injection, and portal vein injection to circumvent pulmonary lodging of transplanted cells and subsequent embolus formation is invasive and often lethal. For these reasons, systemic infusion of MSC-CM represents an effective alternative for delivering the therapeutic effects of MSCs in acute liver injury. Systemic infusion of MSC-CM is a reasonable and effective procedure for delivering the protective effects of MSCs in RILD in *in vivo* models. However, systematic proteomic analyses combined with fractionation studies of MSC-CM are necessary to identify not only the key therapeutic components, but also any potentially harmful secretory products.

In conclusion, systemic MSC-CM therapy has profound inhibitory effects on SEC death and inflammatory response modulation, and it ultimately improves VOD in rats undergoing radiation-induced liver injury. Furthermore, MSC-CM has direct anti-apoptotic effects on cultured SECs, which possibly involve activation of the Akt and ERK cell survival signaling pathways. These findings highlight a potential new therapeutic avenue for protecting against RILD.

## FUNDING

Funding to pay the Open Access publication charges for this article was provided by the Specialized Research Fund for the Doctoral Program of Higher Education of China (20120071110065) and the Research Fund from the Shanghai Municipal Science and Technology Committee (14140902303, 12XD1401800).

## Supplementary Material

Supplementary Data
