# Supplementary material for: Mesenchymal stem cell–conditioned medium prevents radiation-induced liver injury by inhibiting inflammation and protecting sinusoidal endothelial cells
Source: J Radiat Res. 2015 Jun 11;56(4):700–8. doi: 10.1093/jrr/rrv026 (PMC4497399; doi:10.1093/jrr/rrv026)
Supplement: Supplementary Data [file supp_56_4_700__index.html]

Mesenchymal stem cell–conditioned medium prevents radiation-induced liver injury by inhibiting inflammation and protecting sinusoidal endothelial cells — Mesenchymal stem cell–conditioned medium prevents radiation-induced liver injury by inhibiting inflammation and protecting sinusoidal endothelial cells — Supplementary Data 

# Mesenchymal stem cell–conditioned medium prevents radiation-induced liver injury by inhibiting inflammation and protecting sinusoidal endothelial cells

## Supplementary Data

Supplementary Data

- Supplementary Table - pdf file
