# Supplementary material for: Mesenchymal stem cell–conditioned medium prevents radiation-induced liver injury by inhibiting inflammation and protecting sinusoidal endothelial cells
Source: J Radiat Res. 2015 Jun 11;56(4):700–8. doi: 10.1093/jrr/rrv026 (PMC4497399; doi:10.1093/jrr/rrv026)
Supplement: Supplementary Data [file supp_rrv026_rrv026supp_table.pdf]

Primers used for real-time PCR.

Target	Primer sequence
TNF-α	5'-GCTGTCGCTACATCACTGAACCT-3'<br>5'-AATGACCCGTAGGGCGATTA-3'
IL-1β	5'-TCGGCAAACCTAGTGTGCTATG-3'<br>5'-AGACTGCCCATTCTCGACAAG-3'
IL-6	5'-TCGGCAAACCTAGTGTGCTATG-3'<br>5'-CCCATCGACAGGATATATTTTCTGA-3'
Il-10	5'-AATAAGAGCAAGGCAGTGGAG-3'<br>5'-TGTATGCTTCTATGCAGTTGATGA-3'
β-actin	5'-TACAACCTCCTTGCAGCTCC-3'<br>5'-GGATCTTCATGAGGTAGTCAGTC-3'
